# Vital NETosis vs. suicidal NETosis during normal pregnancy and preeclampsia

**DOI:** 10.3389/fcell.2022.1099038

**Published:** 2023-01-05

**Authors:** Florence Guillotin, Mathieu Fortier, Marie Portes, Christophe Demattei, Eve Mousty, Eva Nouvellon, Eric Mercier, Mathias Chea, Vincent Letouzey, Jean-Christophe Gris, Sylvie Bouvier

**Affiliations:** ^1^ Department of Haematology, University Hospital, Nîmes, France; ^2^ Department of Gynecology and Obstetrics, University Hospital, Nîmes, France; ^3^ Department of Biostatistics, Public Health and Innovation in Methodology, Nîmes University Hospital, Nîmes, France; ^4^ UA11 INSERM—UM Institut Desbrest d’Épidémiologie et de Santé Publique (IDESP), Montpellier, France; ^5^ Faculty of Pharmaceutical and Biological Sciences, Montpellier University, France; ^6^ Department of artificial polymers, Max Mousseron Institute of Biomolecules, CNRS UMR 5247, Univ Montpellier, Montpellier, France; ^7^ I. M. Sechenov First Moscow State Medical University, Moscow, Russia

**Keywords:** vital NETosis, suicidal NETosis, neutrophil, pregnancy, preeclampsia

## Abstract

**Background:** NETosis occurs in the context of infection or inflammation and results in the expulsion of decondensed DNA filaments called NETs (Neutrophil Extracellular Traps) into the extracellular environment. NETosis activates coagulation and contributes to the thrombotic risk of inflammatory diseases. To date, two mechanisms of NETosis have been identified: suicidal NETosis, in which neutrophils die after expelling the filaments; and vital NETosis, in which expulsion appears without altering the membrane. Human pregnancy is associated with a mild pro-inflammatory state, which is increased in the event of complications such as preeclampsia (PE). NETosis has been observed in these situations, but the mechanism of its production has not yet been studied. The aim of our study was to evaluate the balance of vital vs. suicidal NETosis in normal pregnancy and in PE.

**Patients/Methods:** Neutrophils from healthy volunteers were stimulated with plasma from normal pregnancies (n = 13) and from women developing preeclampsia (n = 13). Immunofluorescent labelling was performed to determine the percentages and origin of NETs in both groups. Inhibition with suicidal or vital NETosis inhibitors was also performed to validate our results.

**Results:** We found a significant increase in NETs in women with PE compared to women with normal pregnancies. We showed that vital and non-vital NETosis are present in normal and preeclamptic pregnancies. We demonstrated that the higher proportion of NETs observed in PE was due to non-vital NETosis whose main component is represented by suicidal NETosis.

**Discussion:** These results suggest the important part of non-vital NETosis in the pathophysiology of PE.

## Introduction

Preeclampsia is a placenta-mediated pregnancy complication (PMPC) that is still associated with a significant mortality and morbidity rate. It occurs in 5% of pregnancy and is defined by maternal hypertension associated with proteinuria after 20 weeks of pregnancy and/or evidence of maternal acute kidney injury, liver dysfunction, neurological features, hemolysis or thrombocytopenia, or fetal growth restriction (Brown et al., 2018; ACOG, 2019). The pathophysiological mechanism of this complication is still poorly understood. However, it is known to increase the risk of venous thromboembolism (Egan et al., 2015).

Neutrophils are major components of innate immunity. In 2004, Brinkmann et al. (2004) discovered a defence mechanism called “NETosis”, a process that generates the release of histone-rich DNA filaments and granular proteins from the neutrophil into the extracellular domain, including elastase (NE) and myeloperoxidase (MPO) (Urban et al., 2006; Urban et al., 2009; Papayannopoulos et al., 2010; O’Donoghue et al., 2013; Dwyer et al., 2014). The aim of this mechanism is to trap bacteria and avoid their dissemination. However, in the event of an inflammatory phenomenon such as preeclampsia, neutrophils will also produce “neutrophil extracellular traps” (NETs), whose components will have a pro-coagulant and pro-thrombotic activity (Gupta et al., 2005; Yipp and Kubes, 2013; Vayne et al., 2017; Burgener and Schroder, 2020).

So far, two types of NETosis induction mechanisms have been described, involving two distinct metabolic pathways. The first one involves the induction of NADPH oxidase following the activation of protein kinase C (PKC) *via* the ERK-MEK signalling route (Rodríguez-Espinosa et al., 2015; Burgener and Schroder, 2020). This pathway leads to an increase in reactive oxygen species (ROS), generating the destruction of nuclear, granular and cytoplasmic membranes (Papayannopoulos et al., 2010). Translocation of the neutrophil elastase into the nucleus also occurs, leading to the inactivation of histones, thus allowing chromatin de-condensation and the production of NETs (Fuchs et al., 2007; Papayannopoulos et al., 2010; Metzler et al., 2011; Metzler et al., 2014). This mechanism provokes the death of neutrophils, hence the term “suicidal NETosis”.

The second pathway requires different stimuli, including Toll-Like Receptors (TLRs) (Yipp et al., 2012; Pieterse et al., 2016), which *in fine* activate PAD4 (Douda et al., 2015; Naffah de Souza et al., 2018), a protein responsible for the hypercitrullination of nuclear histones. This leads to their inactivation and the de-condensation of chromatin (Wang et al., 2009). The NETs thus created can be ejected into the circulation by a mechanism that is still currently unclear. This involves the destruction of the nuclear membrane and vesiculation which, together, cause the release of NETs into the circulation without destroying the cytoplasmic membranes, hence preserving the neutrophils’ integrity. This mechanism is therefore described as “vital NETosis” (Pilsczek et al., 2010; Yipp et al., 2012; Gupta et al., 2018).

The presence of NETs in pregnancy is now the subject of several studies and has been well demonstrated (Gupta et al., 2005; Sur Chowdhury et al., 2016; Hu et al., 2018; Bouvier et al., 2020; Bouvier et al., 2021). However, several mechanisms seem to co-exist for their establishment and, in the case of preeclampsia, they appear to be exacerbated by other biological processes related to the pathophysiology of this pregnancy complication. During pregnancy, the proinflammatory state itself is an activator of NETosis, as is the release of syncytiotrophoblast microparticles (STBMs) and the human chorionic gonadotropin (hCG), oestrogen, progesterone, and granulocyte colony-stimulating factor (G-CSF) hormones (Gupta et al., 2006; Gupta et al., 2007; Giaglis et al., 2016). In preeclampsia, in addition to the amplification of the inflammatory phenomenon and increased release of STBMs, we also observe the activation of NETosis by IL-8 and a strong hormonal imbalance. Ultimately, it is highly unlikely that all these mechanisms activate a single type of NETosis (Hahn et al., 2012; Giaglis et al., 2016; Konečná et al., 2018).

Based on the existing literature devoted to NETosis in pregnancy and preeclampsia, as well as vital and suicidal NETosis, we felt compelled to analyse the existence of a balance between suicidal and vital NETosis in pregnancy, and to evaluate a potential deregulation of this balance in preeclampsia in favour of a preferred mechanism of NETosis in pre-eclampsia.

## Methods

### Participants

Plasmas of this ancillary study were obtained from a prospective cohort study conducted at the department of Gynaecology and the Haematology Outpatients Department, Nîmes University Hospital (France). Participants were included from June 2015 to December 2018 (“Grosspath” study. clinicaltrials.gov identifier: NCT 01736826). The sample-size estimation was based on the primary objective to compare plasma nucleosomes and cfDNA levels between women with normal pregnancies and women developing placenta-mediated pregnancy complications. Participants were recruited as previously described (Bouvier et al., 2020)*.* Briefly, in the “GrossPath” study, we recruited 30 healthy, non-pregnant volunteers (Group HV), 50 pregnant women with normal pregnancies (Group NP), and 14 pregnant women who had developed preeclampsia (Group PE).

The healthy, non-pregnant women were recruited after a general call to all hospital staff. Ages of controls were matched with the ages of pregnant women. Exclusion criteria were: pregnancy and postpartum period (up to 3 months after childbirth), any history of thrombotic events, chronic disease, chronic or recent infection and cancer.

The 50 pregnant women who finally experienced normal pregnancies were recruited during their initial consultation at the department of gynaecology and obstetrics in their 12th week of gestation. These women gave a single blood sample at the time of delivery. Exclusion criteria were: any previous placenta-mediated pregnancy complications, any history of thrombotic events, chronic disease, chronic or recent infection and cancer. Any patients from Group NP who subsequently developed pregnancy complications during follow-up were then transferred to the abnormal pregnancy group and analysed in that group.

A total of 14 pregnant women who had developed PE were included at the onset of the disease. Among these 14 patients, one woman from Group NP was transferred following the occurrence of preeclampsia in the seventh month of pregnancy. Blood samples were obtained in order to compare biological markers between Group NP and Group PE just before delivery to avoid a bias caused by labour and delivery between the two groups. PE was diagnosed according to the international criteria (Brown et al., 2018; ACOG, 2019)*.* If 24 h-urine collection was not available, the protein-creatinine ratio was measured from a random urine sample (Price et al., 2005).

All plasmas available from preeclamptic women (n = 13/14) were tested for this study. One plasma from the GrossPath study could not be tested due to insufficient quantity. In parallel, 13 plasmas from normal pregnancies were tested. The 13 women with normal pregnancies were selected *via* the propensity score method with maternal and gestational ages as matching variables (+/−2 years old and+/−1 week of gestation respectively).

The main and ancillary studies (clinicaltrials.gov identifier: NCT 01736826 and NCT 05470712, respectively) were approved by the Institutional Review Board (IRB) and ethics committee (IRB no. 22.07.01) at Nîmes University Hospital and by the local Committee for the Protection of Persons undergoing Biomedical Research (CPP Sud Méditerranée III). This clinical investigation was performed in accordance with the Helsinki declaration of 1975 as revised in 1996. All participants had given written informed consent to participate in the trial (NCT 01736826) and to constitute a biobank to be used later for the same theme. All pregnancies were singletons.

The patients’ clinical data—age, smoking habits, blood pressure, information about current and previous pregnancies, delivery, and perinatal outcomes—were all collected.

### Isolation of neutrophils

PMN cells from three healthy consenting volunteers were immediately purified after blood collection using density gradient centrifugation according to the manufacturer’s instructions (Polymorphoprep™, Proteogenix). A complete blood cell count was made in parallel. At the end of purification, 4.5 *10^5^ purified PMNs were seeded on 0.001% poly-L-lysine coated coverslips in a 24-well plate using Roswell Park Memorial Institute medium (RPMI) with 50% phosphate buffer saline (PBS) Ca^2+^, Mg^2+^ without fetal bovine serum (FBS).

### Neutrophil staining and stimulation

For each experiment, several conditions were tested. Purified neutrophils were non-stimulated or stimulated with either plasma from non-pregnant women (negative controls), phorbol-12-myristate-13-acetate at 25 nM, (PMA, Merck Biodevelopment, France, chemical NETs inductor, positive controls (Urban et al., 2009; O’Donoghue et al., 2013; Douda et al., 2015) or plasma from participants (1:1 dilution in RPMI with 50% PBS, Ca^2+^, Mg^2+^ without FBS): normal pregnancy (Group NP) or women having developed PE (Group PE) for 4 h at 37°C as described by Hu et al. (2018).

PMNs were then fixed on coverslip using 4% paraformaldehyde (Santa Cruz Biotechnology, INC) for 10 minutes and stained after three washes with PBS. After permeabilization with PBS/Tween-20 0.2% for 10 minutes, non-specific antigenic PMN sites were blocked with PBS/Tween 0.1% with bovine serum albumin (BSA) 1% without EDTA for 30 minutes. After three additional washes, cells were incubated with antibodies: Fluorescein isothiocyanate (FITC) anti-myeloperoxydase antibody (mouse anti-human clone 5B8, BD Biosciences™, United States, 1:10 dilution); uncoupled anti-citrullinated histone H3 antibody (Rabbit polyclonal to Histone H3 (citrulline R2 + R8 +R17), Abcam™, Canada, 1/1,000 dilution) overnight at 4°C in the dark. Cells were then washed with PBS and counterstained with Alexa Fluor-568 (Donkey anti-rabbit, Abcam™, Canada, 1/2000 dilution) for 30 minutes at room temperature in the dark and, afterwards, with Fluoroshield mounting medium (Abcam™, Canada) containing blue-fluorescent Di Aminido Phenyl lndol (DAPI). Immunofluorescence microscopy was performed on an Olympus BX 60 microscope (CytoVision® 7.5. software, Leica Biosystems). For each condition, six fields evenly distributed on the coverslip were analysed. NETs were identified as elongated extracellular DAPI-stained DNA fibers associated with myeloperoxydase using FIJI software as described by Brinkmann et al. and Arpinati et al. (Brinkmann et al., 2004; Arpinati et al., 2020).

NETs from vital NETosis were characterized by a positive DAPI staining and citrullinated histone H3 (CitH3): MPO+, CitH3+ (Erpenbeck et al., 2016; Arpinati et al., 2020). NETs from non-vital NETosis were deducted from the difference between total NETs (DAPI+) and NETs from vital NETosis (MPO+, CitH3+). Image analysis was performed blindly, without knowledge of the cell-processing conditions.

### Inhibition of vital or suicidal NETosis

The same experiments as described previously were performed using diphenyleiodonium (DPI), a ROS pathway inhibitor before stimulation, at 10 μg/L, 1 h, at 37°C and 5% CO_2,_ to inhibit suicidal NETOsis (O’Donnell et al., 1993; Douda et al., 2015).

As vital NETosis mainly passes through the TLR2 and four pathway (Clark et al., 2007; Semeraro et al., 2011; Erpenbeck et al., 2016; Muñoz-Caro et al., 2021), we also performed the same experiments with anti-TLR2 and anti-TLR4 blocking antibodies (ANTI-HU CD284 HTA125 and ANTI-HU CD282 TL2.1 FG, Life Technologies SAS) at 5 μg/mL for 1 h at 37°C, 5% CO2.

The inhibitors were added to the culture medium in the wells with purified neutrophils before stimulating with plasma.

For each experiment using DPI or blocking antibodies, a non-stimulated well was prepared with dimethylsulfoxyde (DMSO), the solvent present in inhibition reagents, as negative controls.

### Statistical analysis

Statistics were analysed with the GraphPad statistical software program (version 6.07. San Diego, United States) using a non-parametric Wilcoxon test for paired data and the non-parametric Mann-Whitney test for unpaired data. When multiple comparisons were performed, a Bonferroni correction was applied. Quantitative data were expressed as medians and interquartile ranges. Qualitative data were expressed as absolute number and frequency (%). A *p*-value <0.05 was considered statistically significant.

## Results

### Population

Twenty-six participants were analyzed in the study: 13 pregnant women with PE in Group PE, 13 pregnant women without complications in Group NP with maternal and gestational ages as matching variables (+/−2 years old and+/−1 week of gestation respectively) using the propensity score method.

**FIGURE 1:**
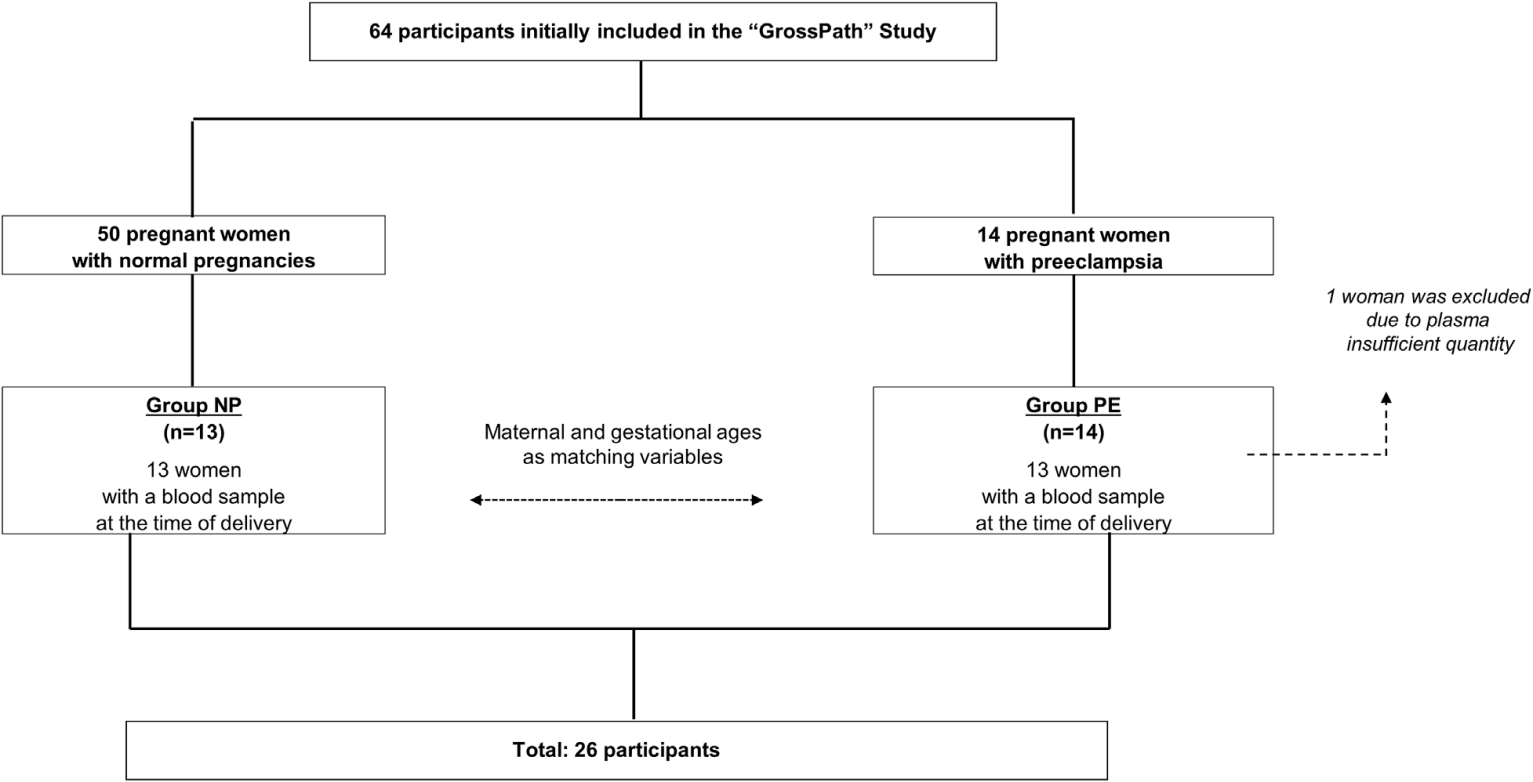
Participant flowchart.

The flowchart is detailed in Figure 1. The characteristics of the population are summarized in Table 1.

**TABLE 1:** Population characteristics N: number; med: median (Q1, Q3): interquartile range; PE: preeclampsia. IUGR: intrauterine growth restriction. NP: pregnant women with normal pregnancies.

	NP	PE	*p*
*Maternal characteristics*			
n	13	13	
Age (years) med (Q1,Q3)	33 (24, 35)	31 (28, 38)	0.99
Active Smokers n (%)	4 (30.8%)	0 (0%)	0.096
Early-onset PE n (%)	−	5 (38.5%)	
Late-onset PE n (%)	−	8 (61.6%)	
Previous pregnancies n (%)	7 (53.8%)	10 (76.9%)	0.41
Previous complicated pregnancies n (%)	1 (7.7%)	4 (30.8%)	0.48
Type of previous pregnancy complications n (%)			
PE	0 (0%)	2	
IUGR	0 (0%)	1	
Stillbirth	0 (0%)	2	
Placental abruption	0 (0%)	0	
Miscarriage	0 (0%)	2	
Chronic hypertension n (%)	0 (0%)	2 (15.47%)	
Pre-gestational diabetes n (%)	0 (0%)	1 (7.7%)	
Type of pregnancy complications during the study n (%)			
PE	0 (0%)	13 (100%)	
Maternal weight before delivery (Kg) med (Q1,Q3)	65 (60, 81)	88 (80, 107)	0.021
Systolic blood pressure before delivery (mm Hg) med (Q1,Q3)	110 (110, 120)	158 (140, 162)	<0.001
Diastolic blood pressure before delivery (mm Hg) med (Q1,Q3)	70 (60.8, 70)	90 (86.3, 96.3)	0.002
Complete Blood Count (CBC)			
Leukocytes med (Q1,Q3)	12.3 (10.0, 13.3)	14.6 (9.9, 16.4)	0.62
Neutrophils med (Q1,Q3)	9. 9.3 (7.2, 9.8)	11.5 (5.1, 13.0)	0.86
Platelets med (Q1,Q3)	221 (185, 274)	181 (158, 258)	0.231
Haemoglobin med (Q1,Q3)	12.5 (11.5, 13.0)	11.5 (11.3, 12.2)	0.22
*Perinatal outcomes*			
Gestational age at delivery med (Q1,Q3)	39 (37, 39)	36 (34, 37)	0.008
Caesarean delivery n (%)	2 (15.4%)	8 (61.5%)	0.044
Neonate weight (g) med (Q1,Q3)	3150 (2860, 3356)	2340 (1740, 2500)	0.0035
Admissions to neonatal intensive care n (%)	0 (0%)	3 (23.1%)	0.22

### The percentage of NETs in preeclampsia is greater than in normal pregnancy

**FIGURE 2:**
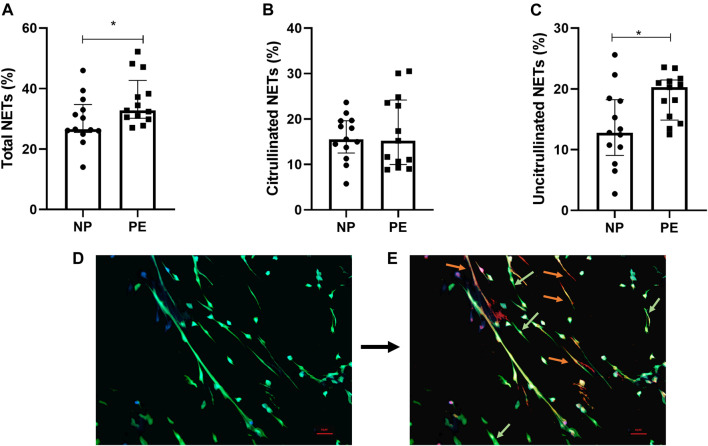
Percentage of total NETs **(A)**, citrullinated NETs **(B)** and uncitrullinated NETs **(C)** formed after stimulation of healthy volunteer neutrophils with plasma from pregnant women delivering a normal pregnancy (NP) and plasma from women delivering with preeclampsia (PE). **(A)** Total NETs from NP, 26.54% [25.48; 34.70]; from PE 32.79% [30.17; 42.70]. **(B)** Citrullinated NETs from NP, 15.56% [12.52; 19.63]; from PE, 15.23% [9.97; 24.20]. **(C)** Uncitrullinated NETs from NP, 12.80% [9.05; 18.23]; from PE, 20.26% [14.87; 21.47]. Results are represented with their medians and interquartile ranges [Q1; Q3] *p < 0.05. Photos taken by fluorescence microscopy with the Olympus BX 60 microscope (Cytovision® 7.5. software, Leica Biosystems), magnification ×20. **(D)** DAPI (blue filter) marking DNA and anti-myeloperoxidase antibody (green filter) marking the granular content of neutrophils. Visualization of total NETs. **(E)** Addition of anti-Histone citrullinated antibody (orange filter) labelling histone hypercitrullination induced by vital NETosis. Differentiation of NETs from vital NETosis (orange) from NETs from suicide NETosis (green).

We found a significant increase in the number of total NETs in women with PE compared to women with normal pregnancies *p* = 0.03 (Figure 2A).

### Vital NETosis and suicidal NETosis are present in normal and preeclamptic pregnancies

Following these results showing the formation of NETs with plasma from pregnant women with normal or preeclamptic pregnancies, we evaluated the different types of NETosis mechanisms implicated in these situations.

We first compared citrullinated NETs, corresponding to vital NETosis, between normal and preeclamptic pregnancies. We did not find any significant difference between the two groups (*p* = 0.96) (Figure 2B).

Then, interestingly, our results showed a significant difference between the number of uncitrullinated NETs, corresponding to non-vital NETosis, between women with normal pregnancies compared to women who developed preeclampsia, *p* = 0.019 (Figure 2C).

We therefore detected the presence of two different types of NETosis in the two situations studied (pictures 2D and 2E). We also observed that the greater part of NETosis in preeclampsia was represented by non-vital NETosis.

### Presence of ROS-dependent suicidal NETosis in normal and complicated preeclampsia pregnancy

Suicidal NETosis described in the literature involves the ROS pathway. This NETosis will be called “ROS-dependent suicidal NETosis” for the rest of our study. To characterize this NETosis in our two groups, we first validated the model with a known activator of suicidal NETosis, PMA, in presence or not of a specific ROS inhibitor, DPI.

**FIGURE 3:**
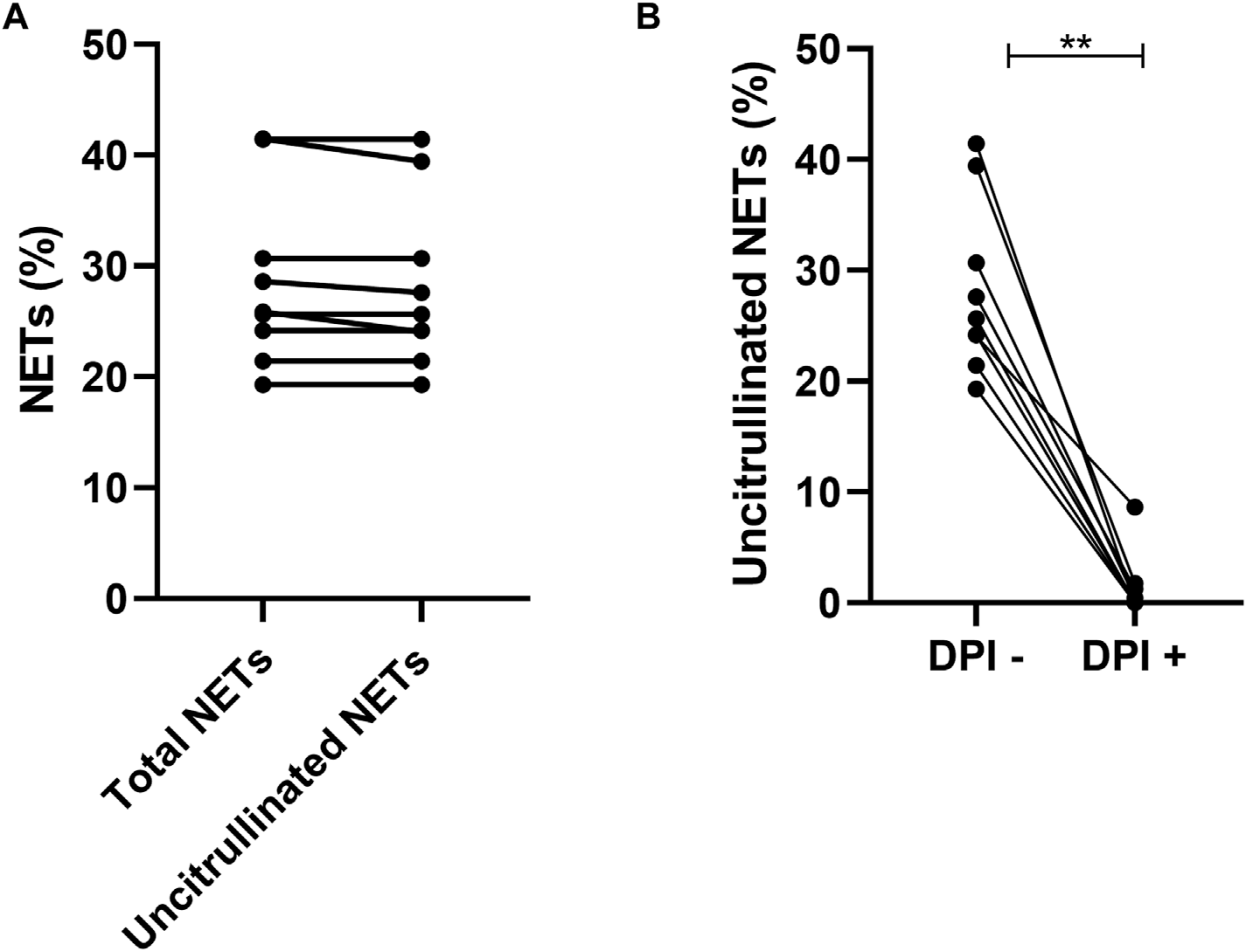
Comparison of total NETs (25.85% [22.83; 36.07]) and uncitrullinated NETs (25.65% [22.75; 35.06]) after induction by PMA *p* = 0.25 **(A)**. Comparison of uncitrullinated NETs after induction by PMA without inhibition by DPI (25.65% [22.75; 35.06]) and with inhibition by DPI (0.00% [0.00; 1.53]) *p* = 0.003 **(B)**. Results are represented with their medians and interquartile ranges [Q1; Q3]. PMA: phorbol-12-myristate-13-acetate. DPI: diphenyleiodonium.**p < 0.01.

NETs from PMA induction were uncitrullinated NETs (*p* = 0.25) (Figure 3A) and this NETosis was completely inhibited by DPI (*p* = 0.0039) (Figure 3B). NETs from ROS-dependent suicidal NETosis may therefore be characterized by uncitrullinated NETs inhibited by DPI.

**FIGURE 4:**
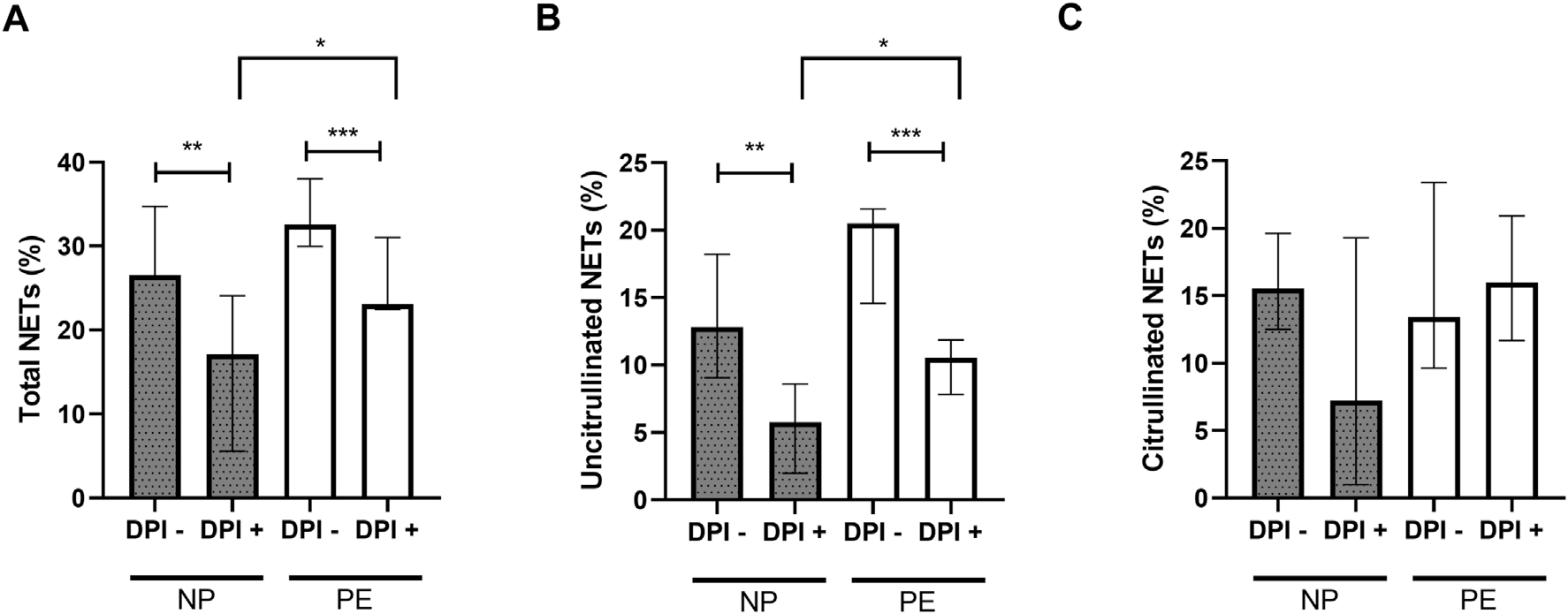
Comparisons of the percentages of total NETs **(A)** and uncitrullinated NETs **(B)** formed after stimulation of healthy volunteer neutrophils with plasma from women delivering normal pregnancies (NP) or preeclampsia (PE) with and without inhibition by DPI. **(A)** NP without DPI 26.54% [25.48; 34.70]; NP with DPI 17.12% [5.86; 24.08]. PE without DPI 32.57% [29.99; 38.01]; PE with DPI 23.08% [22.44; 31.03]. **(B)** NP without DPI 12.80% [9.05; 18.23], NP with DPI 5.76% [1.98; 8.60]. PE without DPI 20.49% [14.58; 21.58]; PE with DPI 10.54% [7.83; 11.87]. **(C)** NP without DPI 15.56% [12.52; 19.63], NP with DPI 7.21% [1; 19.30]. PE without DPI 13.44% [9.63; 23.40]; PE with DPI 16.01% [11.68; 20.93]. DPI-: without inhibition by DPI; DPI+: with inhibition by DPI. Results are represented with their medians and interquartile ranges [Q1; Q3]. DPI: diphenyleiodonium. The Bonferroni correction with a cut-off at 0.025 was applied to take into account the multiplicity of tests. *p < 0.025, **p < 0.01, ***p < 0.001.

The inhibition by DPI of plasma-stimulated neutrophils from the women in our two groups resulted in a significant decrease in total NETs in the NP group (*p* = 0.006) and in the PE group (*p* = 0.001). Moreover, the significant difference in total NETs initially observed between normal and preeclamptic pregnancies (Figure 2A), was maintained in presence of DPI, which meant that total NETs were higher in Group PE than in Group NP (Figure 4A).

The same observation was made with uncitrullinated NETs. There is a significant decrease in the percentage of uncitrullinated NETs before and after inhibition in the NP and PE groups (*p* = 0.0012 and *p* = 0.0005 respectively). Again, the significant difference in the percentage of uncitrullinated NETs initially observed (Figure 2C) was maintained in DPI condition (Figure 4B).

In contrast, this inhibition did not result in a significant decrease in citrullinated NETs, either in Group NP (median without inhibition: 15.56% [12.52; 19.63], median with inhibition: 7.21% [1; 19.30], *p* = 0.15) or in Group PE (median without inhibition: 13.44% [9.63; 23.40], median with inhibition: 16.01% [11.68; 20.93], *p* = 0.34) confirming the link between ROS-dependent suicidal NETosis and uncitrullinated NETs (Figure 4C).

Thus, we confirmed the presence of ROS-dependent suicidal NETosis in normal pregnancy and in preeclampsia.

### Confirmation of the presence of vital NETosis in normal pregnancy and preeclampsia

**FIGURE 5:**
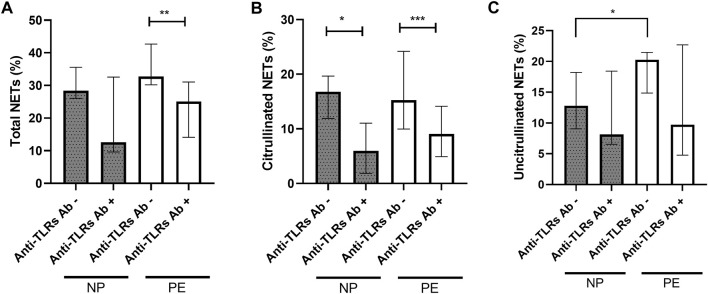
Comparisons of the percentages of total NETs **(A)** and citrullinated NETs **(B)** formed after stimulation of healthy volunteer neutrophils with plasma from women delivering a normal pregnancy (NP) or preeclampsia (PE) without inhibition and with inhibition by anti-TLR2 and TLR4 blocking antibodies. **(A)** NP without anti-TLR2 and TLR4 blocking antibodies 28.40% [26.06; 35.56]; NP with anti-TLR2 and TLR4 blocking antibodies 12.57% [9.59; 32.58]. PE without anti-TLR2 and TLR4 blocking antibodies 32.79% [30.17; 42.70]; PE with anti-TLR2 and TLR4 blocking antibodies 25.05% [14.10; 31.06]. **(B)** NP without anti-TLR2 and TLR4 blocking antibodies 16.77% [11.90; 19.65], NP with anti-TLR2 and TLR4 blocking antibodies 5.96% [1.85; 11.03]. PE without anti-TLR2 and TLR4 blocking antibodies 15.23% [9.97; 24.20]; PE with anti-TLR2 and TLR4 blocking antibodies 9.07% [4.92; 14.13]. **(C)** NP without anti-TLR2 and TLR4 blocking antibodies 12.80% [9.05; 18.23], NP with anti-TLR2 and TLR4 blocking antibodies 8.13% [6.55; 18.43]. PE without anti-TLR2 and TLR4 blocking antibodies 20.26% [14.87; 21.47]; PE with anti-TLR2 and TLR4 blocking antibodies 9.73% [4.79; 22.69]. Anti TLRs+: with inhibition by anti-TLR2 and TLR4 blocking antibodies. Results are represented with their medians and interquartile ranges [Q1; Q3]. The Bonferroni correction with a cut-off at 0.025 was applied to take into account the multiplicity of tests. **p < 0.01, ***p < 0.001.

To confirm the presence of vital NETosis in normal pregnancy and in pregnancy complicated by preeclampsia, we performed experiments using anti-TLR2 and anti-TLR4 blocking antibodies. Inhibition by anti-TLR2 and anti-TLR4 blocking antibodies led to a significant decrease in the number of total NETs in the PE group (*p* = 0.0046 respectively) (Figure 5A). The NP group showed the same trend but with no significance, due to the heterogeneity of the values in presence of TLR antibodies (*p* = 0.043).

It also led to a significant decrease in citrullinated NETs, markers of vital NETosis, in the NP and PE groups (*p* = 0.021 and *p* = 0.0005 respectively) (Figure 5B).

However, this inhibition did not lead to a significant decrease in the number of uncitrullinated NETs either in the NP group (median before inhibition: 12.80% [9.05; 18.23], median after inhibition: 8.13% [6.55; 18.43], *p* = 0.74), or in the PE group (median before inhibition: 20.26% [14.87; 21.47], median after inhibition: 9.73% [4.79; 22.69], *p* = 0.11) (Figure 5C).

There was also no significant decrease after inhibition by anti-TLR blocking antibodies in total NET rates between NP and PE groups, *p* = 0.61 (Figure 5A), in citrullinated NET rates between the two groups, *p* = 0.31 (Figure 5B) or in uncitrullinated NETs *p* = 0.72 (Figure 5C).

These observations confirm the same induction of vital NETosis in normal and preeclamptic pregnancies as previously described.

## Discussion

The major new finding of this study is that both vital and non-vital NETosis are present in normal and preeclamptic pregnancies and that the higher proportion of NETosis observed in preeclampsia is characterized by non-vital NETosis.

Several studies have shown that NETosis process occurs during pregnancy and is more significant when pregnancy is complicated by preeclampsia (Gupta et al., 2005; Sur Chowdhury et al., 2016; Hu et al., 2018; Bouvier et al., 2020; Bouvier et al., 2021). In our study, we first confirmed these two data.

Two main mechanisms of NETosis have been described: vital NETosis and non-vital-NETosis whose main component is represented by suicidal NETosis with no data concerning pregnancy. Therefore, we showed, by immunofluorescent labelling, that both types of NETs, citrullinated from vital and uncitrullinated from non-vital NETosis, were involved in pregnancy and preeclampsia. This labelling also allowed us to identify that the higher proportion of NETosis observed in preeclampsia was characterized by non-vital NETosis. As immunofluorescent labelling to characterize the type of NETosis can be critical due to the subjectivity of the operator (Vayne et al., 2017), we worked under blinded conditions, without knowledge of the cell processing conditions and reinforced our observations by using inhibitors of the different metabolic pathways (anti-TLR2 and TLR4 blocking antibodies for vital NETosis and DPI for suicidal NETosis), before the stimulation with plasma (O’Donnell et al., 1993; Clark et al., 2007; Semeraro et al., 2011; Douda et al., 2015; Erpenbeck et al., 2016; Muñoz-Caro et al., 2021). These inhibitions confirmed the presence of vital and non-vital NETosis, including ROS-dependent suicidal NETosis, in both normal and complicated pregnancies. It also reaffirmed the greater proportion of non-vital NETosis that causes the increase in NETosis in the latest. This observation is in agreement with previous data showing that this pathology is at the origin of an increase in IL-8, a release of cytokines and reactive oxygen species as well as an increase in G-CSF which allows the translocation of neutrophil elastase into the nucleus. These different mechanisms participate in the process of suicidal NETosis (Hahn et al., 2012; Giaglis et al., 2016; Konečná et al., 2018).

However, it is interesting to note that the use of reactive oxygen species inhibitors upstream of stimulation did not result in complete inhibition of non-vital NETosis induced by plasma from women with normal or complicated pregnancies whereas it completely inhibited ROS-dependent suicidal NETosis induced by PMA. This observation highlights that suicidal ROS-dependent NETosis is not the only component of non-vital NETosis and suggests that at least a third metabolic pathway may be involved. Indeed, recent studies have described a suicidal ROS-independent NETosis pathway called non-canonical NETosis (Burgener and Schroder, 2020). This pathway involves inflammasome activation of caspase-4/11 leading to gasdermin D (GSDMD) cleavage resulting in the formation of GSDMD-p30 pores. These pores target the neutrophil membranes to mediate the permeabilization of these membranes, ultimately inducing their rupture. The presence of nuclear GSDMD-p30 pores allows caspase-11 to access chromatin where it exerts a function, like neutrophil elastase, to degrade histones and allow chromatin relaxation (Burgener and Schroder, 2020). Although this pathway was initially described following infection by gram-negative bacteria *via* lipopolysaccharide (LPS), the metabolic pathway of caspase-4/11 and GSDMD is also described in many pathologies with an inflammatory component, notably diabetes (Cheng et al., 2021) but also pulmonary arterial hypertension (Wu et al., 2022) or atherosclerosis (Jiang et al., 2021). As pregnancy, whether normal or complicated by preeclampsia, has an inflammatory component, this newly-described pathway could explain that suicidal ROS-dependent NETosis was not the only component of non-vital NETosis in our cohort and required further investigations during pregnancy.

Inhibition by anti-TLR2 and TLR4 blocking antibodies did not lead to a total inhibition of vital NETosis either. This is surely because this metabolic pathway can be activated by other stimuli such as the complement system or activated platelets (Yipp et al., 2012; Burgener and Schroder, 2020). A similar experiment using PAD4 inhibitors simultaneously with targeted inhibition of different stimuli, might make it possible to characterize the role of vital NETosis in normal pregnancy and preeclampsia more precisely.

One limitation to our study is due to the fact that plasma was collected from women at the end of their pregnancies. This induces a potential bias due to the high inflammatory state observed at this period. It would be interesting to repeat the study using plasma from different periods of pregnancy. This exploration would make it possible to study the evolution of NETosis during pregnancy and to evaluate the various metabolic pathways ahead of the appearance of clinical signs of placenta-mediated pregnancy complications. Another limitation is the fact that we did not characterize Netosis induction mechanism(s). Several factors, such as inflammatory cytokines, microvesicles or sex hormones may induce NETosis and are expressed differentially in Preeclampsia (Hahn et al., 2019). It is highly likely that some of these may preferentially induce a specific type of NETosis.

Our study has also several strengths. It is the first one which interested in the different metabolic pathways of NETosis involved in normal pregnancy and preeclampsia. Our study describes for the first time, the important part of non-vital NETosis in the pathophysiology of preeclampsia.

To conclude, our results showed that pregnant women who develop PE have an increase in non-vital NETosis whose main component is represented by suicidal NETosis. Non-vital NETosis might be part of the link between the risk of thrombosis and pregnancy complications. Further explorations are required, including the effect of treatments on the generation of NETs from non-vital NETosis.

## Data Availability

The raw data supporting the conclusions of this article will be made available by the authors, without undue reservation.
